# Antagonistic muscular co-contraction for skilled, healthy piano technique: a scoping review

**DOI:** 10.3389/fpsyg.2025.1386273

**Published:** 2025-05-01

**Authors:** Cobi Ashkenazi, George Waddell, Aaron Williamon

**Affiliations:** Centre for Performance Science, Royal College of Music, London, United Kingdom

**Keywords:** co-contraction, piano technique, human movement, PRNDs, biomechanics

## Abstract

**Aims:**

This scoping review aimed to generate a novel evidence-based model of antagonistic muscular co-contraction (AMCC)’s effects on human movement. The review applies this model to the context of skilled, healthy piano playing to enable advances in pedagogy and research that can aid pianists in developing and maintaining skill and task-related health.

**Background:**

Piano playing is a challenging, complex activity that carries significant risk of playing-related neuromusculoskeletal disorder (PRNDs). AMCC is a contentious, terminologically problematic topic in pedagogical and scientific literature, and has scarcely been studied in relation to piano technique.

**Methods:**

Adhering to PRISMA-ScR guidelines, the review adopted the search terms “co-contraction,” “piano,” “co-activation,” and “antagonist,” consulting 36 aggregated resources and 100 individual journals. After screening, 188 studies published between 1982 and 2021 were included. From these studies, AMCC-related content was extracted, analyzed in relation to piano technique, and categorized. The resultant categories were synthesized into a model representing the characteristics and effects of AMCC in movement.

**Results:**

AMCC is a prevalent, complex, and learnable phenomenon, exhibiting the capacity for both positive and negative effects on performance and health. These effects are highly relevant to the task-specific challenges of skilled, healthy piano playing. AMCC can affect sensorimotor task control, accuracy, efficiency, coordination, internal model generation, proprioception, range of motion, individuation, neuromuscular signal-to-noise ratio, speed, power, stability, task-related injury, pain, and rehabilitation.

**Conclusion:**

The review and corresponding model suggest that AMCC is a fundamental characteristic of human movement with broad and unique effects on sensorimotor task performance, including piano playing. Of the 188 publications reviewed, none were found to have robust methods investigating AMCC in healthy, skilled pianists; this review underpins ongoing research targeting the nature of AMCC in piano technique.

## 1 Introduction

### 1.1 Context and rationale

Piano playing is a complex neuromuscular task that can place extreme demands on the body (Kinoshita et al., 2007). Pianists face the highest risk of injury among musicians (Kaufman-Cohen et al., 2018); these injuries can be termed playing-related neuromusculoskeletal disorders (PRNDs). PRNDs are professional musicians’ largest health concern (Stanhope et al., 2020), negatively impacting wellbeing, playing ability, and career (Kenny and Ackermann, 2015; Silva et al., 2015; Baadjou et al., 2016). Lifetime PRND incidence for pianists is likely >50% (Rotter et al., 2020; Baadjou et al., 2016; Guptill, 2011; Foxman and Burgel, 2006), and effective treatments and prevention are needed, but pianists report a lack of both (Ciurana Moñino et al., 2017). Some PRNDs result from poor technique (Furuya et al., 2006; Allsop and Ackland, 2010), which impedes playing, performing, career development, and wellbeing (James, 2018; Kotani and Furuya, 2018; Ascenso et al., 2018). Optimal piano technique can require decades to develop, a process which itself risks injury or failure (Goebl, 2017).

To ease the process of developing piano technique and to reduce the incidence of PRNDs, an accurate and thorough understanding of the muscle use underlying skilled, healthy piano playing is needed. However, centuries-long controversy about pianists’ muscle activity persists among pedagogues and researchers (Wheatley-Brown, 2011). This review addresses a particular form of muscle activity: antagonistic muscular co-contraction (AMCC) is the simultaneous contraction of functionally paired muscles, tightening a joint (Saliba, 2019). Resolving the prolonged debate around AMCC in piano technique requires developing an accurate model of AMCC’s role in piano technique. This scoping review synthesizes the extant literature to develop a novel theoretical model of AMCC’s effects on human movement.

### 1.2 Research questions

What is the optimal role of antagonistic muscular co-contraction (AMCC) in skilled, healthy piano technique?

How does AMCC relate to skilled, healthy piano playing?

## 2 Methods

This review follows PRISMA-ScR guidelines. CA consulted databases and libraries to identify papers discussing AMCC in the context of aspects of movement related to piano technique, resulting in the inclusion of 188 studies published between 1982 and 2021. These 188 studies were examined and their discussions of AMCC synthesized to create a theoretical model of AMCC’s characteristics and effects.

### 2.1 Data sources

This review gathered data from 36 aggregated resources (search engines/libraries/research repositories) and 100 individual journals. Supplementary Material 1 provides a complete list of the consulted resources.

### 2.2 Search strategy

The below search terms (and their grammatical variations, e.g., ‘antagonistic’) were selected as they pertain to AMCC and piano technique.

- Co-contraction
- Piano
- Co-activation
- Antagonist

To cover a broad range of sources, no date limits or other filters were employed.

### 2.3 Systematic review protocol

**Figure 1:**
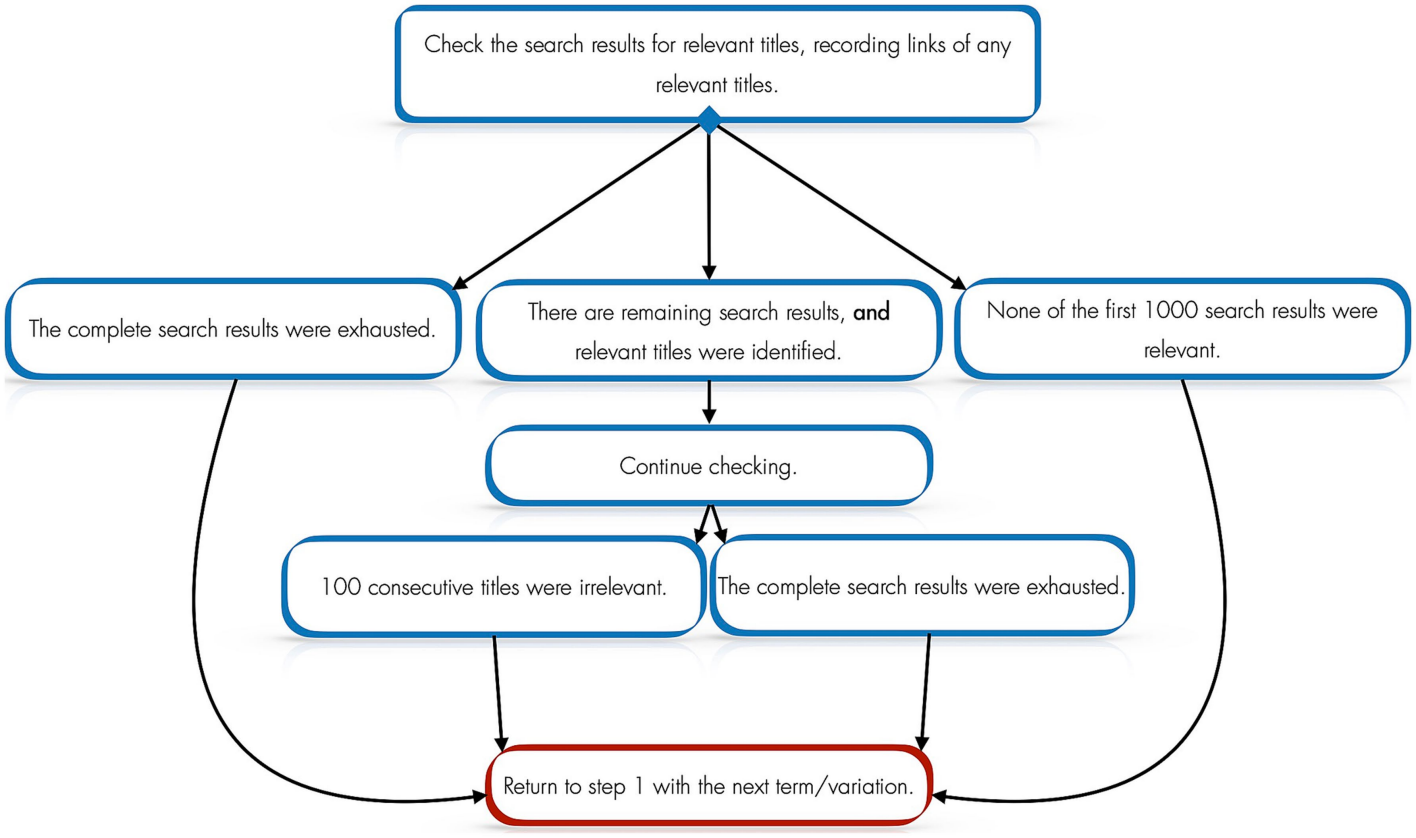
Search process for each resource.

For each resource, CA used the systematic search process outlined in Figure 1.

**Figure 2:**
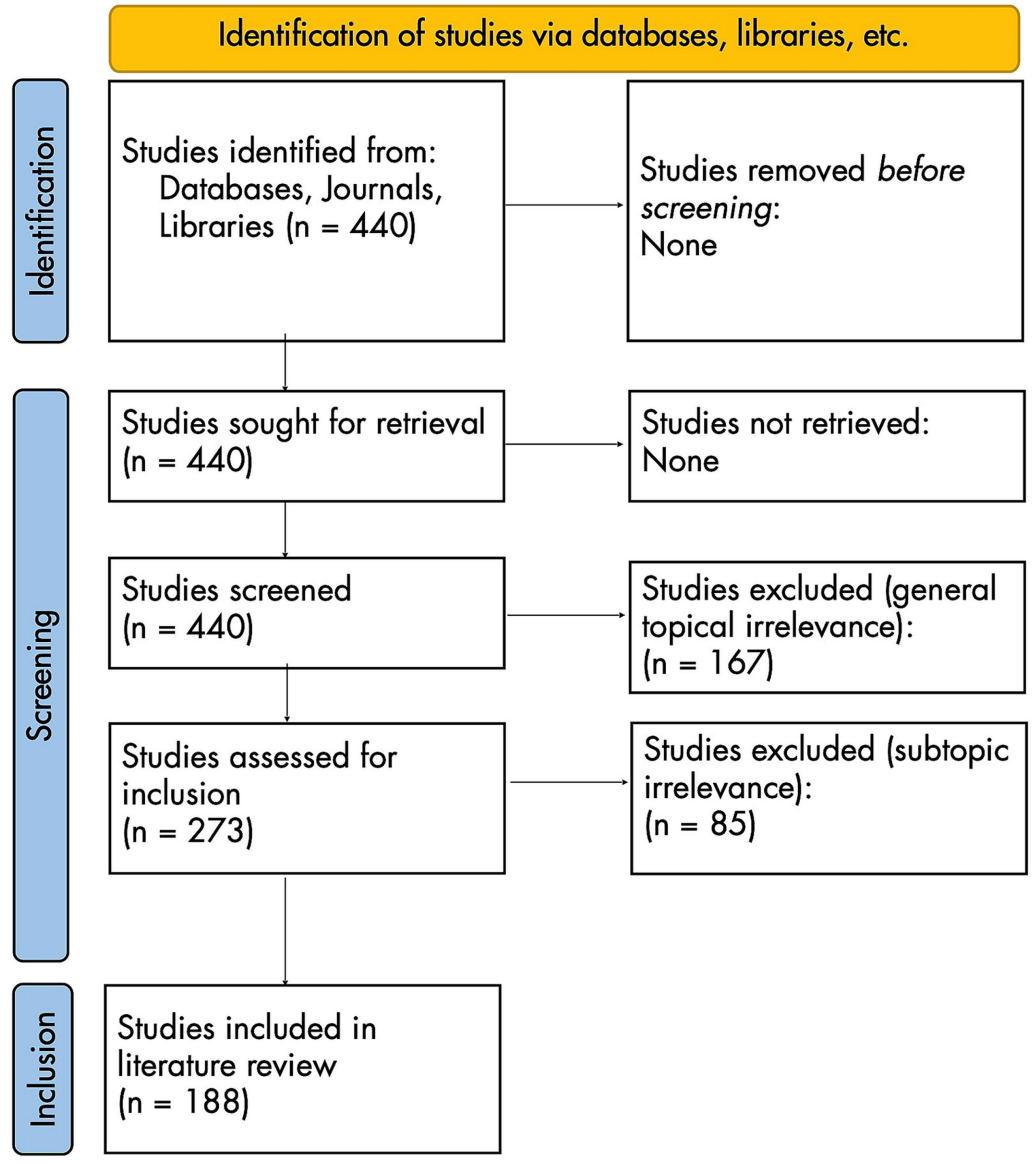
PRISMA 2020 flow diagram for new systematic reviews [adapted from Page et al. (2021)].

This process generated 440 initially relevant titles. Full texts were acquired for all 440, which were then processed according to the adapted PRISMA flow diagram in Figure 2.

### 2.4 Eligibility criteria

English-language articles containing at least one sentence discussing AMCC in relation to aspects of movement pertaining to piano technique were included. For example, an article discussing AMCC’s effects on elbow movement would qualify as indirectly relevant to piano technique, because playing the piano involves elbow movement.

Of the 440 initial articles, 167 were excluded because of general topical irrelevance: these articles’ full texts lacked discussion of both AMCC and piano technique. This left 273 articles, 85 of which were excluded because of subtopical irrelevance: lacking discussion that could be plausibly linked to the relationship between AMCC and piano technique. For example, Parlitz et al. (1998) studied dynamic finger forces in pianists but made no mention of AMCC, focusing instead on absolute measurements of force (Newtons/N).

### 2.5 Data extraction and data analysis

All 188 remaining articles were included. CA read each full text and extracted any discussion pertaining to AMCC. Rather than analyzing quantitative data, this scoping review focuses on points of discussion, concept-formation, and definition, aiming to generate a broad theoretical model of AMCC’s characteristics and effects.

The extracted discussions were reviewed and synthesized according to the following. Commentary was made for each extract, in order of appearance within each article. Each commentary explored potential significance of the extract, considered it alongside the accompanying commentaries in the review, and tentatively categorized the extract according to its conceptual underpinning. For example, Frey-Law and Avin (2013) proposed that AMCC displays “ubiquitous occurrence across activities” (p. 578); this was categorized into the subheading ‘Prevalence of AMCC’. Some articles contained multiple extracts that qualified for more than one conceptual subheading, or one extract that qualified for multiple subheadings; these were discussed anew in each instance.

CA then reread all extracts and commentaries, finalizing categorizations between interrelated extracts and generating groupings of related categories. This process synthesized findings across the 188 articles into a model of AMCC’s characteristics and effects.

The Results section is therefore structured according to this conceptual framework, which is fully visualized at the end of the review as both a tiered list and as a map.

## 3 Results

The analytical process described above produced a 34-point framework illustrating characteristics of AMCC that have been explored in published research. Due to the large number of articles reviewed (188), it was necessary to condense the results here by omitting discussion of articles that made similar points to other articles. Additionally, sections discussing the negative effects, limitations, origins, learnability, and state of knowledge on AMCC have been omitted; further discussion of these can be found in CA’s doctoral dissertation and future publications.

### 3.1 AMCC is prevalent

According to Tubiana and Chamagne (1988), “even the simplest movement requires the participation of antagonists” (p. 86). Morris (2010) found that “co-contraction can be considered an element of all movements” (p. 66–67). Frey-Law and Avin (2013) proposed that AMCC displays “ubiquitous occurrence across activities” (p. 578). Discussing the wrist in piano technique, McCarthy (2016) found that “each movement direction causes co-activation of agonist–antagonist muscle pairs” (p. 26). Goebl (2017) added that “there is certainly no kind of piano technique that eliminates the muscular fixation of joints, be it only for short durations during a keystroke…despite the statements of some piano schools” (p. 3–4). In fact, kinds of piano technique such as the Taubman Approach (Taubman et al., 2005) do eliminate, or at least seek to eliminate, AMCC. Moreover, it bears questioning whether fixation of joints via AMCC only occurs for short durations during a keystroke, given its numerous demonstrated benefits that apply outside these brief time windows.

Mann (1990) observed that AMCC is “a means of joint impedance control,” and therefore “much more prevalent in the activities of daily living than heretofore reported” (p. 73). This demonstrates how the motor control benefits of AMCC contribute to its ubiquity. The use of ‘prevalent’ here affords greater precision than ‘ubiquity’ would, as it allows for the possibility of absence of AMCC. As far as can be interpreted from the included graphs of muscle activity, it appears that all pianists in Furuya (2012) used AMCC across all trial conditions (p. 10).

Piscitelli et al. (2017) found AMCC in “the healthy elderly…persons with atypical development…patients with neurological disorders…as well as in young, healthy persons performing tasks associated with a difficult postural component” (p. 14–15). Mengarelli et al. (2018) found AMCC “in both healthy and pathological populations” (p. 117). This presence of AMCC across populations supports its prevalence. Sousa (2018) argued that “the analysis of reciprocal and simultaneous patterns of antagonist and antagonist muscle activation is considered a fundamental way of understanding motor function” (p. 168).

AMCC appears prevalent across populations and movements, and there does not appear to be reason to doubt its prevalence in pianists and in piano technique—yet this prevalence is doubted by influential pedagogues and researchers (e.g., Lister-Sink, 2018).

### 3.2 AMCC has positive effects

#### 3.2.1 Motor control and motor learning

##### 3.2.1.1 General

Hasan (1986) observed AMCC’s ability to reduce necessary alterations in central drive, which is “far from obvious” (p. 373); this apparently occurs because greater stiffness prevents “fairly large excursions of the equilibrium position…to accelerate and decelerate the inertia in order of the initial and final conditions to be met” (p. 376). This could justify Heald et al.’s (2018) hypothesis that increased AMCC during dynamic motor learning phases “[ensure] the limb remains close to the target state” (p. 1).

Winters and Stark (1986) observed AMCC in “voluntary and externally-driven oscillation, external impulsive loading, and fast movements performed without external loading” (p. 471), concluding that “co-contraction may be a more important real-time control strategy than feedback control via muscle sensory apparatus for most tasks” (p. 471). Glasscock et al. (1997) mentioned that AMCC “appears to be associated with the performance of tasks which require assurance that they be realized effectively” (p. 59). Imagawa et al. (2013) found that “human postural control is achieved by synergistic co-activation” (p. 430). This postural control is possible because synergistic antagonistic muscles have “various action directions” (p. 430). Imagawa et al. (2013) defined muscle synergy as “patterns of activation among multiple muscles involved in controlling movements” (p. 430); this definition therefore includes AMCC, which implies activation of multiple muscles for controlling movements. Chen et al. (2014) found “novel evidence that the antagonist muscle activation is critical during practice” (p. 1017). Xiong et al. (2015), observing AMCC in infant crawling, noted that AMCC is “important for providing adequate joint stability, movement accuracy and energy efficiency” (p. 2115).

Saliba (2019) observed that “co-contraction is a common strategy when performing difficult or unstable motor tasks” (p. i), listing some benefits of AMCC: “to directly increase the mechanical impedance…to generate greater instantaneous restoring forces when a limb or joint is perturbed” (p. i), and also, “the ability to engage both the stretched and shortened muscles in the corrective response” (p. ii). Therefore, AMCC “engages a unique motor strategy” (p. ii). Saliba (2019) also noted a method of reducing the metabolic cost of AMCC: “selective co-activation can optimize the magnitude, shape, and orientation of endpoint impedance to achieve stability at a lower energetic cost than a uniform increase of co-contraction through the limb” (p. 19). This selectivity contributes to the complexity of AMCC, as AMCC can simultaneously vary across joints for varying purposes.

AMCC’s beneficial and unique effects on dynamic motor learning, oscillation, loading, fast movements, and performance in difficult or unstable motor tasks have clear applications in skilled piano technique, which demands exceedingly high performance on complex motor tasks involving oscillation (e.g., repeated notes or trills), loading (e.g., to produce the forces required for loud playing), and speed (e.g., for fast playing).

##### 3.2.1.2 Accuracy and precision

Karst and Hasan (1987) studied antagonist muscle activity in forearm movements, finding that more antagonist activation occurred than “is required for braking alone” (p. 391). This additional activation caused AMCC that was hypothesized to “increase joint stiffness in order to facilitate more precise control” (p. 400). Laursen et al. (1998) studied the effects of speed and precision demands on shoulder muscle activity during a repetitive task, observing that “high precision demands may call for increased stability, which can be obtained by co-contraction of antagonist muscles. Similarly, high-speed muscle contractions have been shown to elicit co-contraction…co-contraction is normally not accounted for in modeling, which as a result has been reported to underestimate the muscle load” (p. 544). Laursen et al. (1998) found “factors such as increased co-contraction” (p. 544) occurred as precision and speed increased. Bawa et al. (2000) found that “cocontraction of wrist extensor and flexor muscle creates a stable basis for finger flexor and extensor muscles to produce precise finger movements, such as fine manipulation in pinch grip of pressing buttons on a computer mouse” (p. 116). Bawa et al. (2000) also noted that “the contemporary work environment includes…repetitive, rapid movements requiring a high degree of precision” (p. 116–117). Gribble et al. (2003) studied the role of AMCC in arm movement accuracy, finding that “as target size was reduced, cocontraction activity increased” (p. 2396). Gribble et al. (2003) also found that “[t]rajectory variability decreased and endpoint accuracy improved” alongside this increased AMCC, suggesting that “although energetically expensive, cocontraction may be a strategy used by the motor system to facilitate multi-joint arm movement accuracy” (p. 2396). van Dieën et al. (2003) concluded that one function of AMCC “might be to achieve more precise control over the trajectory of lifted weight” (p. 1829). Osu et al. (2004) studied optimal impedance control for task achievement, observing that “without the need for great accuracy, subjects accepted worse performance with lower co-contraction” (p. 1199). Osu et al. (2004) accordingly found that higher AMCC was associated with greater endpoint accuracy. Kursa et al. (2005) studied finger flexor forces during isometric tasks, noting that “[d]uring dynamic flexion, finger flexor and extensor muscles are…co-activated” (p. 2289). Kursa et al. (2005) found that “the rate of fingertip force application did not affect the amount of force generated by the extrinsic finger flexor muscles per unit fingertip force during the experimental task” (p. 2292), in contrast with prior studies, which found that flexor and extensor activity both increase “with increasing movement rate and frequency” (p. 2289). Kursa et al. (2005) concluded that “[i]n our study, it is likely that the fine motor control needed to generate the precise force ramps required high activation levels of intrinsic and extrinsic finger muscles in order to stabilize the finger and control joint torques. Therefore, we observed no additional increase in FDP and FDS forces at the higher rates” (p. 2292). This would explain differing results compared to prior studies. Alternatively, given that participants were priorly untrained in the experimental task, it is possible that excessive AMCC was used during slower trials, which was maintained at higher speeds. This indicates the importance of considering the effects of motor learning in neuromuscular research. Kursa et al. (2005) also theorized other potential explanations for their results, including “difference[s] in experimental techniques” or “motion artifacts” (p. 2292). Kursa et al. (2005) also cited that “[c]o-contraction of all seven finger muscles has been reported during a low force, precision grip task…indicat[ing] that all fine muscles are involved in isometric fingertip force generation, but their individual contributions and roles may vary with force, finger posture, and force direction” (p. 2292).

Selen et al. (2005) studied the effects of impedance modulation on kinematic variability with neuromusculoskeletal modeling, finding that “[i]increasing the impedance through co-activation resulted in less kinematic variability, except for the lowest levels of co-activation” (p. 373). Takei and Seki (2010) noted “coactivation of finger muscles characteristic of grasping movements” in monkeys’ precision control of levers (p. 17042). Observing premotor interneurons, Takei and Seki (2010) found that “inhibitory PreM-INs in finger muscles were silent or suppressed during the precision grip task to enhance coactivation of various intrinsic and extrinsic hand muscles” (p. 17049); this might illustrate a cross-species biomechanical necessity of AMCC for precise movement. Frey-Law and Avin (2013) proposed that AMCC is “an important motor control strategy to improve joint stability and movement accuracy…produc[ing] greater movement accuracy and reduced phase lag to external perturbations” (p. 579). Ueyama and Miyashita (2013) modeled signal-dependent noise, co-contraction, and movement accuracy in reaching tasks, finding that “the strength of co-contraction and joint stiffness increased depending on the required accuracy level” (p. 16). Calas-List et al. (2014) studied aimed limb movements in locusts, noting that “[i]n humans, increased co-contraction of antagonist muscles (and thus joint stiffness) enhances movement accuracy…by filtering out the deleterious effects of signal-dependent noise in the motor command (p. 7509). Calas-List et al. (2014) then stated that “[w]hen making fast, accurate movements, humans prefer a speed modulation strategy to a co-contraction strategy (i.e., they use slower movements, not stiffer ones)” (p. 7509). However, this statement is difficult to interpret; if a movement must be executed at a minimum speed, it is impossible to choose a slower movement, and the stiffer one must therefore be preferred.

Le et al. (2017) postulated that “tasks requiring higher stabilization such as precision placement…or higher levels of controlled movements would require higher coactivation” (p. 15). Dupan et al. (2018) noted that “in a precision grip, all muscles are co-activated, and the muscle activity will increase with force” (p. 225); while it is possible to use a precision grip without co-activating all muscles, the precision of the grip would likely be lesser as a result. Ptashnik (2019) noted that AMCC can enhance “both the accuracy and stability of movements…even when destabilizing dynamics are present” (p. 3). Saliba (2019) found “a clear improvement in the performance of participants when they co-contract during the postural perturbation task” (p. 42). This clear improvement was an increase in performance “of up to 350%…with a median performance improvement of ~100%” (p. 43–44). Such a large improvement in movement accuracy need not come at a high cost, however: “rapid corrective responses are generated without overshoot even at the lowest level of co-contraction (equivalent to 1 Nm in each muscle group)” (p. 57). Berret and Jean (2020) developed an optimal control theory that models AMCC in movement. Simulating pointing movements, Berret and Jean (2020) determined that “a minimal level of co-contraction is indeed required to perform the task accurately enough,” which characterized “a trade-off between effort, speed, and accuracy” (p. 15). Koelewijn and van den Bogert (2021) concluded that “co-contraction was optimal for a subset of the tested tasks with a sufficiently high precision…and difficulty” (p. 9).

Sharma and Venkadesan (2022) observed that “[s]table precision grips using the fingertips are a cornerstone of human hand dexterity,” clarifying that “[p]recision grip, as the name implies, is the precise and stable application of fingertip forces. In this grip style, the fingers are relatively stationary while the fingertips exert force” (p. 1). Sharma and Venkadesan (2022) also proposed that “[i]nstabilities that arise when pushing on surfaces can be categorized as those affecting the tip where the force is applied…or the internal degrees of freedom associated with posture…[t]ip instabilities are particularly severe when a stiff finger or limb makes contact with a rigid surface” (p. 1). Sharma and Venkadesan (2022) also report that “[w]hen feedback control is used to precisely apply tip forces, the fingertip’s position in space may become unstable and start to oscillate, which also destabilizes the applied force…One strategy is to increase the compliance of the finger or limb” (p. 1).

Skilled piano-playing is often also a task intended to be performed “as fast and as accurately as possible” (Berret and Jean, 2020, p. 15) while requiring “sufficiently high precision” (Koelewijn and van den Bogert, 2021, p. 9). The above research has explored the beneficial effects of AMCC on precision and accuracy, implying its value as an aid to precise, accurate piano technique, with its own “precise finger movements” (Bawa et al., 2000, p. 116), “repetitive, rapid movements” (p. 116), and small “target size” (Gribble et al., 2003, p. 2396).

##### 3.2.1.3 Efficiency

Roebroeck et al. (1994) studied biomechanics during sit-to-stand transfer, finding that “co-contraction of hamstrings and rectus femoris in sit-to-stand transfer was judged to be efficient” (p. 235). This efficiency of AMCC casts doubt on claims that AMCC is inefficient merely due to its metabolic cost. Instead, AMCC can be seen as efficient in situations where the benefits it affords outweigh the accompanying metabolic expenditure, which follows the same logic as for non-AMCC metabolic expenditures. Roebroeck et al. (1994) commented on this distinction, stating that “in light of required joint displacements, co-contraction of a pair of antagonistic muscles can be judged as inefficient. However, this is a paradox…two antagonistic muscles[,] instead of opposing each other, may reinforce one another by using the tendon action of the other muscle” (p. 242). Zakotnik et al. (2006) studied co-contraction in aimed limb movements in locusts, finding that “co-contraction simplified load compensation” (p. 4995). Without co-contraction, “the extensor would need to generate 16-fold more torque” to move a loaded tibia against gravity (p. 5006). Oliveira and Sanders (2017) studied knee action phase and AMCC during swimming, stating that AMCC “provides dynamic joint stabilization and movement efficiency by tonically stiffening a given joint without impeding net joint torque” (p. 83). Although it is possible that AMCC can impede net joint torque if the desired agonist activation is higher than the antagonist activation subtracted from the maximum potential agonist contraction, the statement that AMCC can improve movement efficiency seems to call into question Yamakawa et al.’s (2017) claim that AMCC is inefficient. Koelewijn and van den Bogert’s (2021) modeling of AMCC found that “even when it is possible to have no co-contraction, it requires less effort to have feedforward control and thus co-contract both muscles” (p. 9). Koelewijn and van den Bogert (2021) further added that “effort is minimized when an antagonistic muscle pair co-contracts” (p. 14), helping to resolve long-standing questions about whether AMCC is efficient; even in a purely mechanical sense, it is seen here that AMCC is required for optimal efficiency, and therefore does not always constitute a waste of energy. Koelewijn and van den Bogert (2021) state clearly that “co-contraction, contrary to what is often thought…is efficient, and…is not chosen out of necessity…but also because it minimizes effort of movement in systems with uncertainty” (p. 15–16). Finally, Koelewijn and van den Bogert (2021) added that AMCC “is often thought of as inefficient and therefore avoided as much as possible” (p. 18), summarizing many authors’ statements on co-contraction, but then concluded that “training and rehabilitation should focus on removing the cause of co-contraction to increase movement efficiency, instead of removing co-contraction itself” (p. 18); this statement potentially undervalues situation-agnostic benefits of AMCC, even those mentioned in the same study. For example, Koelewijn and van den Bogert’s (2021) mention of “noise…present internally in sensory and motor neurons” (p. 3) qualifies as a situation-agnostic for which AMCC apparently corrects.

Efficiency of movement is frequently discussed in piano technique pedagogy (Gerig, 2007), and in these discussions AMCC is indeed “often thought of as inefficient” (Koelewijn and van den Bogert, 2021, p. 18). Given AMCC’s contributions to movement efficiency noted here, its value in efficient piano technique needs careful appraisal.

##### 3.2.1.4 Inter- and multi-joint coordination and transference

Hogan (1984) hypothesized that “as the hand, forearm, and trunk are in series, a high mechanical impedance of the coupling between object and hand would be of little value in providing support for the object if it were not accompanied by a corresponding high impedance between hand and forearm, forearm and arm, arm and shoulder, and so on” (p. 688). Roebroeck et al. (1994) discussed AMCC in sit-to-stand transfer, observing that “biarticular muscles have the better leverage at the joint on which they act as extensor” (p. 242). In the case of sit-to-stand transfer, “the almost isometrically active rectus femoris transports moment from hip to knee joint,” aiding the transfer (p. 243). Furuya et al. (2007) found “strong [AM]CC of the shoulder” when playing faster (p. 40), even though the pianists were playing repeated notes and therefore would not have to reposition their arm around the keyboard. The increased AMCC observed in Furuya et al. (2011) “should allow for a greater transfer of momentum from the limb to the key,” allowing proximal joints to provide greater assistance to the fingers, reducing “peripheral muscle fatigue” (p. 11). Lauer et al. (2013) explored AMCC in front crawl swimming, noting “the importance of the elbow stability in transmitting forces from the hand and the forearm to the body,” which “requires joint stiffness” (p. 820). In piano playing, the same might be true, in reverse: the importance of transmitting forces from the body to the forearm and hand implies the importance of elbow stability, which “requires joint stiffness” (p. 820). Kim and Han (2016) theorized that variation in AMCC might occur due to “angular momentum transferred from the thigh” (p. 6). Huber et al. (2017) observed that “modulating limb impedance [via AMCC] allows humans to coordinate complex, multi-joint movements…during physical interactions and tool use” (p. 3053). Mengarelli et al. (2018) commented that “co-activation of GAS [gastrocnemius] and thigh muscles is recognized as a fundamental mechanism for both stabilizing the knee joint and reducing the reliance on [the] ACL” (p. 118). O'bryan et al. (2018) explored knee extensor fatigue in cyclists, finding a “lack of change in co-activation” during fatigue, which acted as an “inter-muscular coordination strategy…to limit the impact of knee-extensor fatigue on maximal power production” (p. 7). Verdugo et al. (2020) explored trunk and upper-limb factors in the production of loud piano tones, finding that “pelvis and thorax motion can modify both upper-limb linear velocities and joint contribution to generate velocities at the hand and fingers” (p. 20). The use of these proximal body segments to affect more distal segments would seem to require the use of AMCC to allow force to pass through the joints in the kinematic chain; however, Verdugo et al. (2020) did not explore AMCC, only theorizing that pianists might use “muscle co-activation at specific joints to support the keystroke impact and, therefore, to effectively apply the desired effective mass on the keys,” and also that pianists might be able to effectively “push the key downward if an adequate level of joint stiffness (muscle co-activation) is created at the finger joints” (p. 16).

Contrary to piano pedagogues suggesting that AMCC is fatiguing (e.g., Taubman et al., 2005), the findings above suggest that AMCC is an important component of energy-efficient movement, a common topic in piano technique pedagogy. Efficiency is often seen as important in piano technique because of challenging compositions that contain highly taxing and repetitive passages; pianists attempting to play études (e.g., those of Frédéric Chopin or Franz Liszt) must play efficiently so as not to experience muscle fatigue during even a single étude, let alone during the performance (or practice) of an entire set of études.

##### 3.2.1.5 Internal model generation

Heald et al. (2018) stated that “in addition to improving kinematic accuracy, muscle co-contraction also increases the rate of acquisition of an internal model” (p. 8). This faster acquisition rate could be associated with the improved kinematic accuracy, as “any intervention that increases the overlap between the actual motions experienced and the motion required to reach the target, such as increased muscle co-contraction, could increase the rate of adaptation. Second, error sensitivity is greater for smaller errors. This could explain why muscle co-contraction accelerates adaptation, despite decreasing the size of errors” (p. 9). It was also hypothesized that “it is possible that error sensitivity is a function of muscle co-contraction, such that as muscle co-contraction increases, single-trial adaptation is maximized by progressively smaller errors” (p. 9). Heald et al. (2018) found that AMCC “simultaneously [enhances] present and future motor performance,” and that “the modifiable nature of muscle co-contraction suggests that the rate of motor adaptation can be actively modulated” (p. 9). Piano technique, as a form of ‘motor performance,’ is likely to be similarly enhanced by AMCC, as it should also benefit from internal model acquisition, kinematic accuracy, error sensitivity, and decreased error size (e.g., improved adaptation to wrong notes during learning of a new piece of music).

##### 3.2.1.6 Proprioception

Park et al. (1999) explored proprioception in control of goal-directed movement, claiming that “muscle spindles seem to be the primary source of sensory input about changes in joint position and velocity” (p. 631). Park et al. (1999) continued that “[w]ith an active contraction of muscles, muscle spindles become very sensitive to the irregularities in the speed and range of joint movement” (p. 633). This could suggest that AMCC aids proprioception; Morris (2010) claimed that “multidirectional stiffness, such as that seen in tonic co-contraction” can better process afferent information from the body (p. 67). Itaguchi and Fukuzawa (2012) studied the effect of arm stiffness on position reproduction errors, finding that “both constant and variable errors were larger in the direction of lower stiffness rather than in the direction of higher stiffness,” and concluding that “proprioceptive accuracy and precision are positively related to the axis length of elliptically represented arm stiffness, and that exerting muscle effort to maintain the arm against the force of gravity may be supportive of human proprioceptive mechanisms” (p. 757). Itaguchi and Fukuzawa (2012) added that “as stiffness increases, resistance against signal-dependent noise or perturbations of external force also increases. Subsequently, motor commands from the CNS are realized more accurately and precisely in the external workspace” (p. 768). Additionally, Itaguchi and Fukuzawa (2012) explained that “afferent signals from muscle spindles largely contribute to position perception…the sensitivity of muscle spindles increase[s]…accompanied by muscle co-contraction” (p. 770). These mechanisms explain how higher muscle stiffness accomplished through AMCC contributes to improved proprioception. Craig et al. (2017) concluded that “increased co-contraction in older adults is not dependent on contemporaneous proprioceptive input” (p. 3), but this statement appears to discount the contribution of co-contraction itself as a form of proprioceptive input, even though Craig et al. (2017) acknowledged that co-contraction “may be used to increase proprioceptive information” (p. 3). As such, statements like “high muscle co-contraction during the reintroduction of veridical proprioceptive input” (p. 3) risk underemphasizing the potential of co-contraction as veridical proprioceptive input itself, which appears inadvisable given that one of Craig et al.’s (2017) stated purposes is to test whether co-contraction is used to “increase proprioceptive information from muscle spindles” (p. 3). Babadi et al. (2021) found that functional connectivity between the cerebellum and inferior parietal lobule (IPL) was correlated with AMCC, as “one of the functions of IPL appears to be the integration of multisensory information, such as vision and proprioception, in the context of spatial attention and guidance of hand movements” (p. 5674). These findings give a neural basis to AMCC functioning as a proprioceptive aid.

Skilled piano playing demands a refined sense of proprioception; this is evident from the successful performance of compositions with spread-out or quickly leaping hand positions (such that the pianist cannot visually monitor the positions of both hands at once), and additionally from the exceptional abilities of pianists with impaired or no vision (for example, Van Cliburn International Piano Competition winner Nobuyuki Tsujii, who is unable to see due to microphthalmia).

##### 3.2.1.7 Range of motion and individuation

Fujii et al. (2007) studied wrist co-contraction during wrist extension, finding that “co-contraction of PT [pronator teres] and ECR [extensor carpi radialis] during wrist extension movements occurs to prevent supinating the forearm” (p. 80). This AMCC was necessary to prevent supination because of “cross-connections between the distal tendons of ECRL [extensor carpi radials longus] and ECRB [extensor carpi radials brevis]” (p. 80). These cross-connections “pull the distal end of the radius via the retinaculum in supination direction,” a function which had not yet been discussed in prior publications (p. 87). This suggests that, in any situation demanding wrist extension without simultaneous forearm supination, a particular form of AMCC is required. Valero-Cuevas (2005) explored the biomechanical function and neuromuscular control of the fingers, claiming that “co-contraction is necessary to reach most regions of FTS [feasible torque space]” (p. 681). The FTS is important as “to be versatile, the finger should be able to produce net joint torques in all quadrants of torque space” (p. 681). For example, given a particular hand position, there are positions of the fingertip which can only be reached via co-contraction of finger flexors and extensors; to produce joint torques in these spaces therefore demands AMCC. Dupan et al. (2018) studied neural control of hand muscles during single finger pressing, finding that “[i]ntrinsic muscles exhibited individuation, where the agonistic and antagonistic muscles associated with the instructed fingers showed the highest activation.

Given that skilled piano playing requires highly independent, simultaneous movement, positioning, and control of the fingers (particularly evident in the contrapuntal fugues of J.S. Bach, but even required for the playing of a simple C Major chord, and additionally for playing the individual notes of said chord more loudly or softly than one another), AMCC’s importance for aiding individuation and range of motion seems relevant to piano technique.

##### 3.2.1.8 Signal-to-noise ratio (SNR)

Osu et al. (2004) found that, “when subjects were asked to increase co-contraction, the variability of EMG and torque both increased, suggesting that noise in the neuromotor command increased with muscle activation…[yet] the effect of this noise on the task performance is reduced” (p. 1199). Meulenbroek et al. (2005) found that AMCC “forms a strategic means to adapt the flow of motion to central information processing demands” in fine motor tasks (p. 331). Notably, in a handwriting task, “during pen-tip acceleration, co-contraction was clearly higher in the between-letter connection strokes than in the within-letter strokes” (p. 345). Meulenbroek et al. (2005) observed that AMCC is “a likely mechanism to slow down movements in complex motor tasks…whenever increased cognitive demands have to be coped with,” acting “as a low-pass filtering mechanism to increase the signal-to-noise ratios of neuromotor signals when these signals happen to be impoverished by increased task demands or conditions of physical, emotional, and/or psychosocial stress” (p. 347). These contexts have clear parallels with varying aspects of piano playing, from learning new repertoire, practicing known repertoire, and stressful performance situations.

The increase in AMCC observed in Ueyama and Miyashita (2013) was thought to improve accuracy by “reducing the perturbing effects of joint-interaction torques,” and additionally by “suppress[ing] the influence of increased motor noise as a result of rising motor command” (p. 16). Reeves et al. (2016) claimed that “the central nervous system increases muscle activation to account for less precise motor control, possibly to improve the responsiveness of human motor control” (p. 166). Reeves et al. (2016) also noted that increased AMCC “helps minimize the effects of neuromuscular noise” (p. 172). This appears to add a layer of complexity to Fitt’s law, which claims “there is a trade-off between speed and accuracy for self-directed movements” (p. 172), because “humans appear capable of moving faster while maintaining accuracy by increasing agonist–antagonist muscle activation” (p. 173). While there are clearly limits on the extent to which speed and accuracy can be dually maintained, and limits on each variable in isolation as well, skilled piano playing appears to be an example of how AMCC can be used as a strategy to partially circumvent Fitt’s law. Koelewijn and van den Bogert (2021) studied AMCC’s potential in minimizing effort in uncertain situations, hypothesizing that “co-contraction is optimal in practice, due to noise in the movement…present internally in sensory and motor neurons” (p. 3). Koelewijn and van den Bogert (2021) added that “human control should constantly correct any deviations caused by noise…[but] a neural time delay is present in the control due to the travel time required through sensory and motor neurons. Therefore, “the stiffness added by co-contraction prevents any unwanted deviations due to noise” (p. 3). AMCC’s ability to prevent noise-based movement deviations could be especially valuable for piano technique, as small errors of positioning or movement can quickly compound to interruptions in playing, particularly during complex and fast passagework. Even a single interruption in playing can have consequences during a live performance, particularly in compositions involving other musicians (for example, piano concerti or chamber music), also increasing the risk of a lapse of context-dependent memory (a ‘memory slip’) due to the physical interruption (Mishra, 2002).

##### 3.2.1.9 Speed and power output

Stanier (1973) found that “the antagonistic muscles should be tensed prior to the movement” if it is desired to move a limb “as quickly as possible once only through a given straight line” (p. 4–28). Stanier (1973) does not explain why AMCC is required to create the fastest possible movement, but it could be related to differing timings of muscle contraction and relaxation, or preparatory joint stiffness increasing the efficiency of the following contraction. However, Stanier’s (1973) discussion of “rotational pressure transfer” (p. 9–13) neglects the benefits that AMCC might provide, and therefore advises that “the antagonistic muscles of these joints could also be applied to give a degree of stiffness, and so for that matter could the finger muscles, but theoretically this is not necessary” (p. 9–10). Barnes (1982) explored the role of pre-stimulus AMCC in elbow flexion movements, finding that “movement times and average angular velocities were significantly improved after pre-stimulus antagonistic contractions were performed” (p. 678). Williams and Barnes (1987) found a positive correlation between elbow AMCC with both “movement velocity and displacement…indirectly support[ing] the notion that the antagonist musculature provides a braking force to arrest rapid limb movements” (p. 933). Gabriel et al. (1993) found that “decrease in movement time associated with practice was accomplished by an increase in the slope of the agonist/antagonist EMG bursts” in ballistic elbow movements (p. 327). Given that piano playing requires elbow extension (Furuya and Kinoshita, 2008), often at higher velocities, this might imply the need for elbow AMCC in pianism.

Rouard and Clarys (1995) studied AMCC in maximal-effort swimming, finding that “cocontractions are important features of rapid cyclic repetitive movement” (p. 177). Rouard and Clarys’s (1995) findings “suggested the presence of cocontraction through different parts of the range of the cyclic arm motion” (p. 182), and concluding that “cocontractions were an important aid in rapid, cyclic, rhythmic and repetitive movement performance” (p. 183). Pezarat-Correia et al. (1996) found that AMCC “seems to represent more than an impulse braking…we must admit its participation in the control of the end of the acceleration phase” of fast throwing movements (p. 486). This AMCC could “control movement time,” or “improve the performance of rapid elbow movements…providing a longer time for acceleration and an increase in movement velocity” (p. 487). Furuya et al. (2007), observing highly trained pianists, found “increases in joint angular velocity and co-contraction (CC)… of all upper limb muscles before keystroke” when dynamics, measured in SPL (sound pressure level), were increased (p. 40). Furuya et al. (2007) concluded that “by increasing joint stiffness and movement speed, the pianists increase the amount of the momentum transfer from the hand into the key” (p. 40). Furuya et al. (2007) found “strong [AM]CC of the shoulder” when playing faster (p. 40), even though the pianists were playing repeated notes and therefore would not have to reposition their arm around the keyboard. The AMCC observed in Furuya and Kinoshita (2008) “was clearly larger at a larger sound for both groups of players,” suggesting that greater AMCC could be required for loud playing (p. 588). This does not, however, imply a linear relationship between loudness and optimal AMCC. Larsen et al. (2008) noted that “[a]ntagonist muscle force exertion plays an important role in the execution of fast ballistic limb movements in order to make a rapid transition from joint flexion to extension” (p. 578). Andison (2011) studied wrist AMCC in piano playing, finding “significant variations in co-contraction that corresponded to faster note rates and increased loudness” (p. iii). Andison (2011) concluded that “the presence of co-contraction is fundamental to piano playing” (p. iii).

Latash (2018) noted how AMCC prevents delays arising from the distance and speed of CNS-muscle communication: “it can take over 100 ms before neural processes produce visible changes in muscle activation. This is a very long time delay, potentially incompatible with successful performance of everyday motor tasks, such as standing, which rely on quick reactions to unexpected perturbation” (p. 99). AMCC is also advantageous “if the task is to move as quickly as possible,” or (in systems with fixed origin, such as piano playing) “if the task is to improve task stability” (p. 100). Schwalbe et al. (2019) explored red muscle activity in bluegill sunfish, finding that “fish co-activated anterior muscle…to stiffen their bodies during acceleration” (p. 2). Schwalbe et al. (2019) noted a trade-off between how “[f]ish with more flexible bodies should…use less energy to swim steadily,” yet fishes’ “bodies should be stiffer for rapid, impulsive movements” (p. 3). Saliba (2019) found that “co-contraction reduces the overshoot of the return to target by between 0.7 cm and 1.7 cm compared to when the agonist is stretched or shortened” in a reaching task (p. 75). A single centimeter can be the difference between a correct or incorrect note on the piano keyboard. Additionally, “co-contraction also reduces the return time to the target at all levels of background activity” (p. 75); this could have implications for passages in piano music that require moving rapidly between different hand positions (e.g., Franz Liszt’s *Réminiscences de Don Juan*, S. 418).

The findings of this review so far provide thorough underpinning to Andison’s (2011) claim that “co-contraction is fundamental to piano playing” (p. iii). Because AMCC is seen as essential to a diverse range of mechanisms underlying human movement, its value in piano technique should not be dismissed – despite the insistence of anti-AMCC pedagogues past and present (e.g., Matthay, 1908; Stannard, 2014).

##### 3.2.1.10 Stability (general)

Bautmans et al. (2012) stated that AMCC “might be a physiologic compensation…to increase joint stiffness and maintain joint stability” (p. 3), while Lauer et al. (2013) noted that “coactivation is the most robust strategy to counteract perturbations” (p. 820). Lee et al. (2017) noted that AMCC “plays an important role in enhancing joint stability for movement regulation during motor learning activities” (p. 1). Saliba (2019) stated that “the primary benefit of co-contraction is from neural feedback responses,” breaking from prior literature supporting impedance control as the primary benefit of AMCC (p. 111). Saliba (2019) explains that “the effects of neural feedback have been incorrectly characterized as mechanical impedance,” but “that is not to say that the effects of impedance are not significant” (p. 111). It might be possible, however, that the primary benefit of AMCC is context-dependent and differs across varying situations, especially given the communication delays between the CNS and muscles observed by Latash (2018), which would imply impedance as more valuable than neural feedback response in situations demanding instantaneous reaction. Babadi et al. (2021) found that “the cerebellum appears to play the predominant role in regulating co-contraction, as it is “ideally structured to register errors in motor commands…and, therefore, to implement countermeasures such as co-contraction to counteract external disturbances” (p. 5674).

Given that piano playing involves external disturbances resulting from the equal and opposite reactions of the key against the impact of the fingers via Newton’s Third Law, and the above findings that AMCC appears to have an important role in counteracting external disturbances, it seems that a further function of AMCC in piano technique is the counteraction of these key-reactions, which if not counteracted can interrupt the position and trajectory of the fingers, hand, and even arm.

##### 3.2.1.11 Stability (upper limb)

Stanier (1973), an early doctoral thesis on piano technique, also was possibly “the first time that piano playing has been studied with a computer” (p. 2–2). Stanier (1973) found that prior authors show “almost complete unanimity over the state of antagonistic muscles during rapid oscillation…[both] must be used simultaneously” (p. 4–15). As to why many authors insist that this AMCC must be of a high intensity for rapid oscillatory movement, Stanier (1973) hypothesized that “perhaps a large amount of antagonistic muscle force is necessary to give stability to the joint” (p. 4–17), a hypothesis borne out by later research. Stanier (1973) continued: “how is it possible for a joint to be made rigid? As each joint is equipped with a pair of antagonistic muscle groups, the answer would seem to be that the muscle groups both switch on and thus work in opposition to each other” (p. 4–26). This is an accurate description of AMCC creating joint rigidity. Hogan (1984) observed AMCC “under normal physiological conditions…increas[ing] as gravitational torques increase” to “offset gravitational destabilization,” increasing stability (p. 688). Hasan (1986) noted that “if joint stiffness were an undesirable property for the performance of movement, one would expect that during self-initiated movement there would be no coactivation of antagonists muscles…[yet] there is considerable evidence for the coactivation of antagonists for part or whole of the duration of many normal movements” (p. 373). This statement argues inductively that joint stiffness, and therefore AMCC, must be necessary. Hasan (1986) found that “seemingly wasteful coactivation may serve to optimize the stiffness. The stiffness, therefore, need not be viewed simply as a means of resisting imposed perturbations, but as a means of reducing the alterations in the central drives necessary for the performance of movement, thereby reducing the effort” (p. 373). This combats claims that AMCC is necessarily energetically inefficient, wasteful, or excessively effortful. De Serres and Milner (1991) found that “cocontraction [increased] dramatically” (p. 451) to increase wrist stiffness when loads were unstable, while phasic stretch reflexes did not contribute to stiffness. Piano playing arguably includes unstable loading, as heavy loads can be transferred to keys through fingers during fast, complex movements.

Gribble and Ostry (1998) studied independent control of shoulder and elbow AMCC, finding that “shoulder muscle co-activation was…independent of the co-activation of elbow and double-joint muscles” (p. 359). Between-joints, independent AMCC can “stabilize the limb in the face of external perturbing forces and forces arising from multipoint dynamics” (p. 355). Damm and McIntyre (2008) advised that “[a]rm stiffness is a critical factor underlying stable interactions with the environment…[and] seems to increase in free space compared with constrained motion through the use of coactivation” (p. 2577). Damm and McIntyre (2008) found that “[the] CNS uses coactivation of antagonists to deal with unexpected external forces when performing unconstrained movements in free space” (p. 2584). Wong et al. (2009) argued that in “highly unstable” environments, “the only functional solution available to the motor system is to produce an increase in limb stiffness,” and therefore suggesting that “it is thus important to address whether the nervous system uses stiffness control to facilitate movement accuracy in more naturalistic tasks that do not involve external destabilizing force loads” (p. 1542). Wong et al. (2009) found that “the motor system uses stiffness control to augment movement accuracy during movement and does so in the absence of external unstable force loads, in response to changing accuracy requirements conveyed using visual cues” (p. 1542). Wong et al. (2009) concluded that “neural control of limb stiffness is an integral part of the voluntary control of movement” (p. 1548). This conclusion has clear implications for piano playing, which itself is a form of voluntary, controlled movement.

Brookham et al. (2011) argued that “Upper limb control and end effector precision depend on effective elbow stability, which makes co-activation especially important at this joint” (p. 1582). Furuya and Soechting (2012) studied independent control of finger movements in pianists, finding “maintenance of independent finger movements across tempi” suggesting speed invariance of these movements in expert pianists, and also that “an increased finger muscular coactivation may enable maintained rhythmic accuracy of keystrokes across tempi,” because “augmented stiffness ensures mechanical robustness against spontaneous variability of motor commands” (p. 2067). Blache et al. (2014) studied co-activation of superficial shoulder muscles during lifting tasks, discovering that “more co-contraction” occurred during the dropping phase compared to pulling and lifting phases (p. 355). This co-contraction was thought to “be a solution to increase glenohumeral joint stiffness” (p. 355). Sangwan et al. (2014) found that “the hypothesis that rotator cuff muscles show co-activation to provide joint stability was partially supported” (p. 7). Additionally, “the maximum EMG amplitudes were in expected directions,” providing further support for the stability theory of rotator cuff AMCC (p. 9). Holmes et al. (2014) observed that “co-contraction increased in all pairs as grip force increased” in perturbed forearm gripping (p. 1). This increase in AMCC corresponded to “a 36% increase in overall wrist joint stiffness,” which aided control of the wrist, especially during perturbations (p. 1). Kawai et al. (2014) simulated AMCC and movement control, noting that “co-contraction of antagonist muscles play an important role for joint stiffness and stability and experimental results show the existence of co-contraction during volitional movements” (p. 3316). Kawai et al. (2014) concluded that “co-contraction is useful…also to control the output force direction,” reducing “tracking errors” (p. 3320). Dupan et al. (2018) found that “activation in both agonistic and antagonistic muscles appears to facilitate finger stabilisation” (p. 224). This AMCC is also likely active in piano playing, as finger stability presupposes wrist stability. Dupan et al. (2018) found that “extrinsic muscles show an activation independent from instructed finger in both agonistic and antagonistic muscles, which appears to be associated with stabilization of the wrist” (p. 224). Rinaldi et al. (2018) studied biomechanics of the Junzuki karate punch, finding that “the upper limb is more stiffer [sic] than both trunk and lower limb in order to generate more powerful movements” (p. 9). Rinaldi et al.’s (2018) analysis of this higher AMCC in the upper limb is difficult to follow, however: “force creates faster movement, but the corresponding stiffness slows the change of muscle shape and joint velocity” (p. 8). How does one force create both faster movement and slower joint velocity? Rinaldi et al. (2018) also stated that “rapid relaxation may be helpful to enhance the speed strength” (p. 9), but it is unclear what ‘speed strength’ entails and the source of this ‘rapid relaxation’ is not specified.

Schinkel-Ivy and Duncan (2018) noted that “external perturbations that are unpredictable, continually changing, and multi-directional” require “both feedforward responses for the oncoming perturbation, and feedback mechanisms for the perturbation at hand. Consequently, an adequate response must be produced for the current perturbation without compromising stability for the oncoming perturbation. These responses require complex patterns of muscle activity” (p. 42). Though perturbations experienced during piano playing are not typically unpredictable, they do qualify as both continually changing and multi-directional, and therefore also are likely to require simultaneous feedforward and feedbackward responses. For example, responding to the current reaction from the keyboard due to playing a note might ideally occur simultaneously with a reaction to the upcoming note(s). Huber et al. (2019) noted that “the challenge of controlling physical interaction arises from the fact that, when you apply forces on an external object, the object simultaneously applies forces back onto you. The object’s dynamics are coupled to your dynamics, and this can destabilize the physically coupled hand-object system” (p. 51). Although ‘controlling physical interaction’ offers more than one challenge, the forces applied in reaction (via Newton’s Third Law) are indeed an important factor in destabilization. Huber et al. (2019) also observed that “extensive prior work suggests that humans are able to ensure robust stability during physical interaction by modulating the mechanical impedance of their limbs,” which occurs via AMCC (p. 51). Snyder et al. (2019) investigated visuomotor control of arm stability, noting that “[d]uring movement, agonist muscles are activated to move the limb toward the target, which is followed by antagonist muscle activation to provide braking” (p. 2156). However, antagonist muscle activation does not only ‘follow’ agonist activation, but also occurs simultaneously, for reasons including braking. Snyder et al. (2019) then clarified that “[i]ncreasing the co-contraction of the arm during arm movements and postural maintenance tasks results in better movement accuracy and less positional error, respectively, providing increased stability to the limb during reach” (p. 2156). Snyder et al. (2019) concluded that “visuomotor control of arm posture involves co-contraction of antagonistic muscles” (p. 2165); given that piano playing requires visuomotor control of arm posture, these findings appear to imply that piano technique requires AMCC.

Forman et al. (2020) found that forearm “[c]o-contraction ratios were higher in the flexion conditions…likely a contribution to wrist joint stability” in dynamic wrist flexion-extension (p. 1). Notably, during extension conditions, co-contraction was still present, with flexors at “only ~32% the activity of the wrist extensors” (p. 7). Sharma and Venkadesan (2022) concluded that “people are significantly cocontracted when producing fingertip forces, likely for stability” (p. 7).

Beyond the external disturbances addressed in 3.2.1.10, the “multipoint dynamics” (Gribble and Ostry, 1998, p. 355) of upper limb movement are a relevant source of internal disturbances when operating complex motor tasks, piano playing being no exception (Kinoshita et al., 2007); this further suggests the importance of AMCC in piano technique.

##### 3.2.1.12 Stability (lower limb)

Nissan (1980), found that “[a]gonistic and antagonistic activity of muscles and loading of ligaments were shown to be possible and helpful in balancing the knee” (p. 375). Hirokawa et al. (1991) noted that “during execution of a specific motion, the agonist muscles supply most of the force needed to accomplish the set objective while the antagonist muscles exhibit low-level activity ranging from 5 to 50% of their maximal force” in cadaver knee stability (p. 199). Hirokawa et al. (1991) continued that “much data supports the hypothesis that such muscular co-contraction is the “modus operandi” in most limb joints and is a significant factor in providing brisk, accurate movement with regulation against various internal and external disturbances such as the changing direction of the gravity vector, motion speed, external load, dynamic braking, muscle movement arm variations, and skill development” (p. 199). Gravitational adjustment offers a further justification for omnipresent AMCC, as gravity is a constant factor in human movement. Hirokawa et al. (1991) concluded that “muscular coactivation serves at least four important physiologic functions; providing brisk precise control of limb motion, allowing development of skill, regulating against various internal and external disturbances, and maintaining joint stability” (p. 207). Hirokawa et al. (1991) also cautioned that “joint stability…could be severely compromised if agonist muscles were the only active actuators during motor tasks” (p. 207), conflicting with anti-AMCC piano pedagogy. Bernardi et al. (1995) found that “when the muscle acts as antagonist most motor units are recruited up to 50% of the maximal voluntary force, whereas when the muscle acts as antagonist motor units are recruited up to 40% of the maximal voluntary force” in knee AMCC (p. 493). However, this statement is self-contradictory; it is likely that ‘agonist’ was meant rather than the first instance of ‘antagonist.’ Bernardi et al. (1995) noted that AMCC can produce “a dynamic braking at the end of the motion,” and that “the importance of muscular coactivation in low-level contractions…is effectively reducing the anterior displacement and the internal rotation of the tibia, preventing excessive stress of the joint by providing synergistic action to the anterior cruciate ligament. The role of the coactivation results in a regulatory stabilizing function…and a more accurate performance of each motor task” (p. 497–498). Kellis and Baltzopoulos (1999) studied antagonistic muscle force during isokinetic efforts in the knee, noting that “hamstrings activation when acting as antagonists is considered very important for knee joint stability” (p. 19). Aagaard et al. (2000) explored AMCC during knee movements, finding “[s]ubstantial hamstring coactivation,” “potentially counteract[ing] the anterior tibial shear and excessive internal tibial rotation…to assist the mechanical and neurosensory functions of the anterior cruciate ligament,” causing “improved stability” (p. 58). Zhang and Wang (2001) discovered that “differential co-contraction of muscles crossing the medial and lateral sides of the knee…helped to reduce the abduction-adduction joint laxity…increase stiffness…and viscous damping” (p. 1107). This knee AMCC increased “natural undamped frequency,” which “presumably makes the neuromuscular system operate more quickly at higher contraction levels” (p. 1114). Additionally, the AMCC and corresponding stability were hypothesized to make the knee “a quicker system during strenuous tasks involving strong muscle contraction” (p. 1107).

Zakotnik et al. (2006) found that “in cockroaches, joint stiffness attributable to co-contraction is a key parameter for running speed and adaption to different surface compliances…and stabilizes the impact when the leg touches ground at the beginning of the stance movement” (p. 5006). Larsen et al. (2008) studied AMCC during stair walking, noting that AMCC “serves to increase joint stiffness and provide protection against external impact forces as well as enhancing the stiffness of the entire limb” (p. 569). Rao et al. (2009) studied the effects of loading on knee muscle activation, confirming “the advantageous role of cocontraction because the contribution of active stiffness to joint stability depends both on the knee angle and on the external load” (p. 464). Rao et al. (2009) also found that “with higher loads, requiring a higher stability of the knee joint, the peak of cocontraction is shifted to an angular range where the efficiency of the hamstrings muscles to actively stabilize the knee is maximal” (p. 464). Di Nardo et al. (2015) assessed ankle AMCC during normal gait, finding “significantly increased complexity in muscle recruitment strategy beyond the activation as pure ankle plantar/dorsiflexors” (p. 347). This strategy “suggests that co-contractions are likely functional to further physiological tasks as foot inversion, balance improvement, control of ankle stability and knee flexion” (p. 347).

Di Nardo et al. (2016) found that “in healthy children co-contractions are likely functional to further physiological tasks as balance improvement and control of joint stability” (p. 161), and “useful to stabilize and smooth the double-to-single support transition” in walking (p. 165). Strazza et al. (2017) found “three different co-contractions among QF [quadriceps femoris] and hamstring muscles during able-bodied walking” (p. 228). These co-contractions were seen to “augment ligament function in maintenance of joint stability, providing resistance to rotation at a joint and equalizing pressure distribution at joint surfaces” (p. 228). Craig et al. (2016) argued that “[o]lder adults use a different muscle strategy to cope with postural instability, in which they ‘co-contract’ the muscles around the ankle joint” (p. 251). This statement could imply inaccurately that younger adults do not co-contract the muscles around the ankle joint to combat postural instability. Craig et al. (2016) found that “despite suggestions from previous research…better proprioceptive acuity predicts more co-contraction” (p. 251). This conflict with prior research might be explained by the fact that AMCC can both increase proprioceptive acuity and also itself be increased in individuals with compromised proprioceptive acuity, perhaps as a result of the former. Given the assumption that AMCC cannot increase proprioceptive acuity to such an extent that it would outweigh any observed impairments in proprioceptive acuity, this might explain the existence both of studies finding AMCC in proprioceptively impaired populations, and of studies such as Craig et al. (2016) finding higher AMCC in populations with higher proprioceptive acuity. However, Craig et al. (2016) concluded that AMCC is not used “to compensate for age-related proprioceptive deficits” (p. 251), stating that “[o]ur findings contradict the recurrent prediction that muscle co-contraction is a compensatory strategy for age-related proprioceptive decline by emphasizing that co-contraction is employed more by older adults with good proprioception” (p. 257). Craig et al. (2016) made this statement despite AMCC’s ability to increase proprioceptive function due to the fact that “no muscle co-contraction was witnessed” during the proprioceptive task (p. 258). The conclusion that AMCC is not a compensatory strategy for proprioceptive decline is questionable: given that AMCC is known to improve proprioception and is “employed more by older adults with good proprioception” (Craig et al., 2016, p. 258), it is perhaps more likely that the particular proprioceptive task, or which muscles’ AMCC was measured, were not conducive to elicitation and/or detection of AMCC. Craig et al. (2016) also theorized that “in everyday life muscle co-contraction is an ineffective and risky postural strategy” because of findings that voluntary AMCC can increase postural sway amplitude and frequency (p. 252). However, this claim seems not to distinguish between necessary vs. excessive AMCC, instead oversimplifying AMCC as an undesirable and simplistic phenomenon. Craig et al. (2017) investigated proprioception, postural sway, and AMCC in older adults, finding that “increased co-contraction in older adults is not dependent on contemporaneous proprioceptive input…it is more likely that cocontraction is a general postural strategy used to minimize postural sway” (p. 2). This could overlook the benefit of AMCC itself improving proprioceptive input. Le Mouel and Brette (2019) advised that “[i]ncreasing ankle stiffness in advance of a perturbation can improve robustness to perturbations, by reducing the amplification of perturbations during the neural feedback delay” (p. 3). Le Mouel and Brette (2019) also noted that AMCC “allows perturbations to be canceled faster, with less overshoot…and less increase in contraction” (p. 8). Jafarnezhadgero et al. (2021) found that AMCC aids “foot inversion, balance improvement, control of ankle stability and knee flexion” for females with genu varus (p. 76–77).

In piano technique, AMCC could similarly aid the upper-limb parallels of these lower-limb functions. Additionally, an easily overlooked and under-researched area of piano technique is the usage of the foot pedals; while pedaling itself is perhaps a simpler task than playing on the foot pedals of a pipe organ, skilled pianists still must maintain balance on the piano bench while accomplishing complex pedaling (Rosenblum, 1993), and the effects of AMCC on lower limb stability seem likely to be relevant for this challenging task.

##### 3.2.1.13 Stability (trunk)

Thelen et al. (1995) found that “co-contraction is a major determinant of spinal loading” in trunk movement (p. 390). This AMCC served multiple purposes: “to stiffen the joint so as to minimize the effect of potential internal and external disturbances…to equilibrate moments at other joints in the case of multiarticular muscles, or…to regulate the loads at the joint” (p. 397). Given that piano playing involves trunk movement, it is possible that trunk AMCC is an important component of piano technique, but it has apparently not been discussed priorly. Choi (2003) found that “neck muscle co-contractions are necessary to provide stability to the human cervical spine around its neutral posture by stiffening the joint” (p. 139). This neck AMCC could be important in piano technique as particularly forceful passages might otherwise perturb the neck and head, which could upset listening, proprioception, and other crucial mechanisms of skilled playing. It appears that neck AMCC has not been discussed in the context of piano technique, except for instructions to avoid “unnecessary tightening of neck muscles” (Lister-Sink, 2018, p. 40); these instructions are potentially counterproductive when not accompanied by definition of *necessary* neck tension. Gribble et al. (2003) noted that AMCC “affects joint impedance, which provides technical stability in the presence of external perturbations and forces due to limb dynamics” (p. 2396). These increases in AMCC were observed “despite the energetic cost of muscle coactivation” (p. 2396). van Dieën et al. (2003) found that “[a]bdominal coactivation was significantly higher” when lifting unstable loads, supporting “the interpretation of abdominal cocontraction during lifting as subserving spinal stability (p. 1829). Lee et al. (2006) determined that “[t]runk stiffness increased 37.8% (*p* < 0.004) from minimal to maximal co-activation” (p. 51), which “empirically validate[d] the assumption used in published models of spine biomechanics that co-contraction influences trunk stiffness” (p. 5). Borghuis et al. (2008) claimed that “a low level of co-contraction of the trunk muscles is important for core stability,” providing “a level of stiffness, which gives sufficient stability against minor perturbations” (p. 894). This attenuation is important so that the CNS can “[create] a stable foundation for movement of the extremities through co-contraction” (p. 894). Borghuis et al. (2008) also noted that “joint stiffness increases rapidly and nonlinearly with muscle activation, so that very modest levels of muscle activity create sufficiently stiff and stable joints” in the context of core stability (p. 896). Additionally, many tasks “could not be performed without this co-activation…result[ing] in stabilization of the excessive mobility of the extremities” (p. 899). Relevant to pianists, “the art, especially for athletes, is to enhance mobility, while at the same time preserving sufficient stability” (p. 899). McCook et al. (2009) studied co-contraction of lumbo-pelvic muscles, noting that “[c]o-contraction of the lumbo-pelvic muscles is required even in neutral upright postures because the passive structures of the spine are inadequate to maintain stability of the lumbar spine” (p. 754). This claim appears to presuppose that stability of the lumbar spine is a desirable goal; it should be possible (though difficult) to remain upright without co-contraction (e.g., by contracting only agonists, balancing against the pull of gravity). However, stability is widely seen as desirable. Murray et al. (2018) found that mice react to external perturbations “by generating a motor program of muscle extension, followed some 30 ms later by co-activation of antagonist muscles in the hindlimb” (p. 1336). In piano technique, perturbations occur as the keys react against the fingers with equal and opposite force due to Newton’s Third Law; however, as these perturbations are not unpredictable, reactive AMCC should not occur 30 ms post-perturbation, but instead, as a preemptory response (Le Mouel and Brette, 2019). Homayounpour et al. (2021) studied neck AMCC during harmless head impacts, finding that preparatory AMCC helped “reduce the kinematic response after the impulsive force to the head,” demonstrating that AMCC is an important mechanism to resist perturbations (p. 4).

Given the importance of stability in complex and difficult movements, the contributions of AMCC to stability across the upper and lower limbs as well as the trunk and neck further imply its importance in piano playing, which itself is a highly complex and difficult form of movement (Kinoshita et al., 2007).

#### 3.2.2 Medical benefits of AMCC

##### 3.2.2.1 Therapeutic

AMCC can aid ligament injuries: O’Connor (1993) found that “simultaneous contraction…can unload the cruciate ligaments entirely at flexion angles above 22°” (p. 410), completely protecting knee ligaments after injury or repair. This AMCC strategy could “help the design of rational regimes of rehabilitation after ligament injury or repair” (p. 410). Lee et al. (2012) found that injured ballet dancers “had greater co-contraction…for the non-dominant ankle” (p. 693), which was thought to “take over the function of damaged ligaments to maintain joint stability” (p. 695). This AMCC was “considered as an efficient mechanism to protect joints against potentially dangerous loads” (p. 695). Gallego et al. (2013) developed a neuroprosthesis which “employed transcutaneous neurostimulation to apply mechanical loads…in co-contraction—in such a way that joint impedance was adequately manipulated” (p. 2), which “constitutes a feasible approach to tremor management…through the control of muscle co-contraction” (p. 7). Notably, “all patients reported that the sensation generated…was tolerable and not unpleasant, and the overall impression was that they could habituate to it…a few patients spontaneously declared that when the [neuroprosthesis] was activated they could control better their limbs” (p. 7). The 100% positive response rate was especially valuable given that “a significant proportion of those suffering from tremor do not respond to medication” (p. 10). This application of neuroprosthetic AMCC stimulation as a viable therapeutic tool, alongside research of AMCC as being therapeutic for other disorders (Overbeek et al., 2018), demonstrates AMCC’s restorative potential. Biscarini et al. (2016) found that “voluntary quadriceps cocontraction…can yield considerable levels of quadriceps activation while preventing the tibia from translating forward relative to the femur,” therefore qualifying as “one of the most appropriate quadriceps strengthening interventions in the early phase of ACL rehabilitation” (p. 1). Biscarini et al. (2016) also noted that “hamstring cocontraction is inherent to natural knee extension and serves important physiological functions, such as stabilizing the tibiofemoral (TF) joint and reducing the mechanical landing of the ACL” (p. 3). Rosa et al. (2014) argued that AMCC is important “for providing optimal joint stability, good movement accuracy and energy efficiency during functional activities” for neurologically impaired individuals (p. 3). Rosa et al. (2014) concluded that AMCC shows strong relationships with “kinematics, dynamic strength, postural stability, walking speed and walking independence in subjects with stroke” (p. 14), also noting that “Similar relationships have been reported in osteoarthritis…cerebral palsy…Parkinson’s disease…and in healthy elderly people” (p. 14). Rosa et al. (2014) also found increased AMCC “during walking after stroke in both the affected and non- affected limb, most likely as an adaptation strategy to increase walking stability” (p. 14), also observing that “[s]lowest walking speeds post-stroke are usually associated with inability to recruit additional MCo [AMCC]” (p. 14). Al-Khlaifat et al. (2016) found that osteoarthritic knee patients displayed increased knee AMCC compared to healthy controls, which “might be a protective mechanism to improve knee joint stability during gait in the presence of muscle weakness with knee OA [osteoarthritis]” (p. 63).

Kolk et al. (2021) found that patients with subacromial pain syndrome (SAPS) used “less antagonistic activity of the teres major” than healthy controls, and that “[m]any authors linked deficits in shoulder muscle activation to SAPS pathogenesis” (p. 16). Resultantly, Kolk et al. (2021) recommend to “increase co-contraction” (p. 19) for SAPS patients. Overbeek et al. (2018) found that increased shoulder AMCC was “associated with a favorable course” in SAPS (p. 1925). Specifically, AMCC of arm adductors during arm abduction helped to significantly reduce complaints of pain, even at a four-year follow-up evaluation. This reduction in pain was thought to be caused by “widening of the subacromial space” (p. 1925), helping to prevent “painful upward migration of the humerus” (p. 1926) due to compromised shoulder stability. This resulted in “significantly increased quality of life…indicating a clinically relevant improvement” (p. 1928). Conversely, “unchanged activation patterns…were associated with persistent complaints” (p. 1928). Yuan et al. (2019) found that rectus femoris AMCC “contributes to the stability of the knee and lower limb function…[and] should be considered in the rehabilitation of knee stability during gait” in hemiplegic stroke patients (p. 7443).

Because of the prevalence of playing-related neuromusculoskeletal disorders (PRNDs) among pianists (Kaufman-Cohen et al., 2018), and the general importance of AMCC in piano technique as proposed in this review, AMCC displays potential as a therapeutic activity in the context of practicing the piano while attempting recovery from a PRND (or indeed from other types of task-related disorders). For this reason, it is especially concerning that much of injury-preventative piano pedagogy recommends against AMCC, especially during attempted rehabilitation (e.g., Mark, 2004). This review provides ample reason to reexamine such recommendations. An important caveat to be discussed in future publications is that *excessive* and/or *static* AMCC can have injurious, rather than rehabilitative or preventative, effects. This is not unique to AMCC, but seems true for any kind of muscular activity or energy expenditure.

##### 3.2.2.2 Preventative

Baratta et al. (1988) cautioned that a “reduced coactivation pattern of the unexercised antagonist…increases the risk of ligamentous [knee] damage” (p. 113), therefore recommending that AMCC could reduce “risk of knee injuries in high performance athletes” (p. 113). Baratta et al. (1988) also noted that AMCC is important to distribute “articular surface pressure…under various loading conditions”; without this AMCC, articular surface separation “will create a focused stress point that will rapidly contribute toward focal deterioration along the articular surface and result in early tissue damage and osteoarthritis” (p. 120). By extension, it is apparent that AMCC could reduce the risk of PRNDs by creating “nearly equal pressure distribution over the complete articular surface contact area,” arrangements which “reduce the overall articular surface pressure, prevent focal surface damages, and elongate the period over which good functional service could be provided by the articular interface” (p. 120). Additionally, in regards to muscle hypertrophic imbalance, “the coactivation pattern of the antagonist to hypertrophied muscle is significantly inhibited and subjects the joint to a high risk of injury. A complementary resistive exercise for the antagonist…may restore the muscular balance and reduce the exposure to ligament injury risk” (p. 121).

Hirokawa et al. (1991) noted that “additional evidence…supports the notion that hamstring antagonist co-contraction serves to relieve strain in the ACL…and also to reduce rotary laxity of the knee significantly” (p. 199), hypothesizing that “hamstrings co-contraction, during knee extension, can prevent anterior or rotary displacement of the tibia and thereby reduce the strain on the ACL” (p. 199). Hirokawa et al. (1991) additionally found that “significant anterior displacement and internal rotation of the tibia occurred during isolated quadriceps loading, whereas significant reduction in anterior displacement and rotation occurred upon simultaneous low-level loading of the hamstrings” (p. 199). Hirokawa et al. (1991) also commented that “voluntary increase in hamstring co-contraction, coupled with the fact that increase in its force increases its effectiveness as a synergist to the ACL, could be used advantageously as effective therapy in ACL-deficient patients” (p. 206). Aune et al. (1995) found that “hamstrings and gastrocnemius co-contraction protects the anterior cruciate ligament against failure” in rats (p. 147). This protection occurred as “the simultaneous contraction of the quadriceps, hamstrings, and gastrocnemius controlling knee flexion may unload the cruciate ligaments entirely” (p. 150). Rothmuller and Cafarelli (1995) argued that AMCC helps to “avoid excessive strain on the joint capsule…safeguard[ing] the agonist muscle against complete exhaustion” (p. 864).

Yeadon et al. (2010) noted that “[i]n landings from a flight phase the mass center of an athlete experiences rapid decelerations” (p. 364). Through observing muscle use during an elite martial artist’s landings with sEMG, and simulating these landings in a computer model, Yeadon et al. (2010) found that “co-activation…was necessary to land successfully from heights greater than 1.05 m” (p. 367), and moreover that this lack of preemptive co-contraction can place the knee in an “inappropriate and possibly dangerous position for landing” (p. 364). Yeadon et al. (2010) suggested that “[t]he same considerations apply in any activity where rapid changes in net joint torque are required” (p. 369). Billot et al. (2014) found that, despite elevated knee AMCC in older males, “antagonist torques were not responsible for age-related declines” in knee joint torques (p. 899). These findings suggest that increased AMCC in aged populations is not a contributing factor to impaired function, instead being “mainly described as a protective mechanism at a joint” (p. 900). Holmes et al. (2014) noted that “uncoordinated muscle actions may lead to wrist joint instability and/or injury,” and that AMCC “aided control of the wrist,” establishing the value of AMCC for healthy movement (p. 1). Ranavolo et al. (2015) developed a new index to evaluate AMCC in work activities, noting that AMCC helps to “provide added protection and avoid LBDs [low-back disorders]” (p. 1).

Shiba et al. (2015) explored electrically stimulated AMCC in an astronaut on the International Space Station, attempting to mitigate disuse muscle atrophy. Shiba et al. (2015) found that electric stimulation of AMCC during exercise had a “preventive effect…on an astronaut’s musculoskeletal atrophy” (p. 12). Le et al. (2017) hypothesized that sedentary work “may require intermittent periods of increased coactivation in order to encourage blood flow and muscular substitution to mitigate myalgia or myofascial pain from prolonged loading” (p. 3). Oliveira and Sanders (2017) found AMCC throughout the eggbeater kick, which was highest in the “final phase of knee flexion” (p. 88–89), which allowed “rapid reversal of the motion from flexion to extension while also stabilizing the joint to prevent injury…requiring increased muscle co-activation” (p. 89). DeMers et al. (2017) found that “strong preparatory co-activation…prevented ankle inversion from exceeding injury thresholds” in landings, while “conversely, stretch reflexes were too slow to generate eversion moments before the simulations reached the threshold for inversion injury” (p. 1). High-force impacts between fingers and keys also pose stability and injury concerns, especially given the physically repetitive nature of pianism.

Sousa (2018) proposed that “assuming the hypothesis that the activity of all muscles within the system is independent…it can be hypothesized that subjects with chronic ankle instability…would present deregulation of antagonist co-activation at the ankle joint level during compensatory postural responses to an external perturbation” (p. 169). Sousa (2018) found “differences between groups for antagonist co-activation…only observed in MLR [medium latency responses]” (p. 171). These responses are considered important because “only MLR responses [sic] have a stabilizing effect during perturbations of stance” (p. 172). Specifically, these differences were “increased antagonist co-activation of MLR of SOL/TA [soleus/tibialis anterior] in a support position and decreased antagonist co-activation of MLR in TA/P [tibialis anterior/peroneus] in the uninjured limb in the support position and in the injured lim [sic] in the perturbed position” (p. 172). However, this “bilateral decrease of antagonist co-activation…would be [sic] probably lead to decreased mediolateral functional ankle stability” (p. 172). Overbeek et al. (2019) explored AMCC in arm movements across age groups, noting that AMCC “may be crucial for counteracting deltoid forces, depressing the humerus and ensuring free passage of subacromial tissues underneath the acromion during abduction” (p. 1). Overbeek et al. (2019) found that middle-aged individuals used more shoulder AMCC than young adults, concluding that “[t]his may indicate that during ageing, alterations in activation patterns are required for preserving pain-free shoulder function” (p. 1). Flaxman et al. (2021) found that “quadriceps hamstring co-activation was associated with knee abduction” in weightbearing (p. 1). These results “highlight the importance of muscular co-activation of all muscles crossing the knee to support it during injury-inducing loading conditions such as externally applied knee abduction and rotation” (p. 1).

Musicians, including pianists, are athletes of the small muscles (Quarrier, 2013); AMCC’s injury-preventative function also imply the importance of developing a piano pedagogy that seeks the proper integration of AMCC in piano technique from the early stages of beginner-level training; this should help reduce the incidence of PRNDs faced by the developing pianist.

### 3.3 Limitations; assessment of risk of bias

Perhaps the foremost limitation of this review is that it omits sections regarding the negative effects, limitations, origins, learnability, and state of knowledge on AMCC, due to limitations of space. These topics are to be discussed in future publications. A second limitation of this review is its English-centric approach. Because only English-language articles were considered for inclusion, this review cannot offer perspective on AMCC and piano technique research outside the English-speaking research sphere; particularly, Chinese-language articles, given the piano’s popularity in China, and steady rises in Chinese-language research output, are a notable omission here. A further limitation of this review is that it was conducted primarily by one individual (CA), though with supervision by GW and AW; CA’s personal understanding of AMCC and his own perspective on piano technique both contributed to the analysis and synthesis of concepts in this review. Nevertheless, we propose that the findings of this review do not depart significantly from the included studies themselves, and furthermore, that the propositions made here regarding the nature of skilled, healthy piano technique (for example, that it requires refined proprioception and efficient movement) are uncontroversial.

## 4 Discussion

### 4.1 Conceptual framework of AMCC, in summary

This review has illustrated the nature of AMCC via its synthesized conceptual framework, which manifested into the following meta-categories:

- General characteristics (section 3.1)
- Positive effects (section 3.2)
- Negative effects*
- Further considerations*

*As noted, the “Negative effects” and “Further considerations” sections are not published in this article due to space considerations, but are to be discussed in future publications.

These categories and their sub-areas represent a 34-point framework suggesting AMCC’s duality and complexity, providing a rich theoretical base for subsequent inquiry into AMCC’s role in healthy, skilled piano playing.

**Figure 3:**
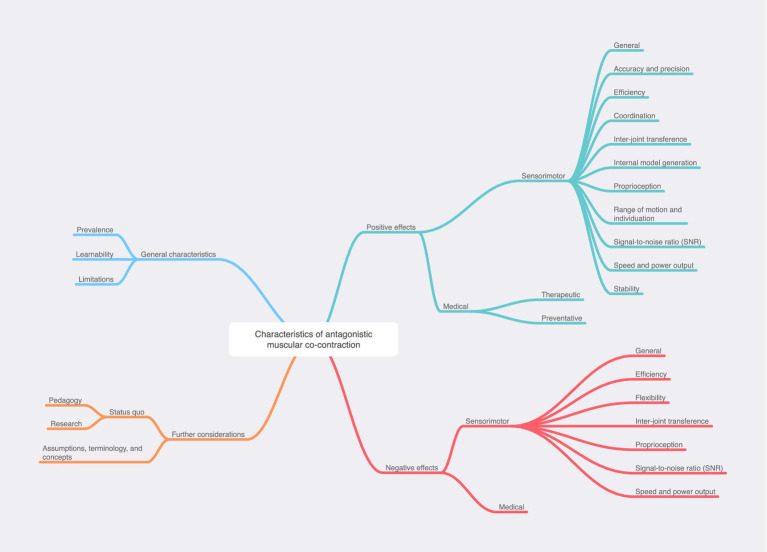
Characteristics of antagonistic muscular co-contraction (network).

Figure 3 depicts the characteristics of AMCC identified by this review.

Given the characteristics and effects of AMCC outlined here, more targeted investigation of AMCC’s roles specifically in piano technique is required to sufficiently fulfill the aims of this inquiry; CA’s doctoral thesis proceeds along these lines. Of the 188 publications included in this literature review, only 14 discuss AMCC in piano technique directly, and of these, a single study (Furuya and Kinoshita, 2008; see section 3.2.1.9) had the requisite methodology and design to begin to directly answer the research questions posed by this review. Table 1 summarizes these 188 publications. As such, the present ongoing research continues to explore AMCC’s characteristics, accounting for its aspects of duality and complexity and handling the set of yet-unaddressed considerations raised in the development of the above framework, which provides a foundation for this further inquiry, synthesizing AMCC’s known characteristics. As such, the findings of the review constitute a valuable step toward situating AMCC in our understanding of healthy, skilled piano technique, which remains a key need for pianists.

**Table 1:** Summary of highest-relevance publications.

	Discusses AMCC in piano technique	Investigates AMCC in piano technique	Focuses on AMCC in piano technique	Real participants	Selected satisfactorily for healthy, skilled pianists	Robust, valid methodology
Furuya and Kinoshita (2008)	√	√	√	√	Partially	√
Furuya et al. (2007)	√	√	√	√	?	√
Andison (2011)	√	√	√	√	No	No
Wheatley-Brown (2011)	√	√	√			
Furuya (2012)	√	√		√	Partially	No
Ortmann (1929)	√	√		√	?	
Yoshie et al. (2009)	√	√		√	?	
Stanier (1973)	√	√				
Furuya and Soechting (2012)	√			√	Partially	No
McCarthy (2016)	√			√	Partially	Yes
Taubman et al. (2005)	√					
James (2012)	√					
Goebl (2017)	√					
Lister-Sink (2018)	√					

## Data Availability

The original contributions presented in the study are included in the article. Further inquiries can be directed to the corresponding author.
