# Supplementary material for: Antagonistic muscular co-contraction for skilled, healthy piano technique: a scoping review
Source: Front Psychol. 2025 May 1;16:1386273. doi: 10.3389/fpsyg.2025.1386273 (PMC12079104; doi:10.3389/fpsyg.2025.1386273)
Supplement: Supplementary file 1 [file Table_1.docx]

| Aggregated resources / search engines / libraries which contained no relevant publications | Individual journals which contained no relevant publications | Individual journals which contained relevant publications | Aggregated resources / search engines / libraries which contained relevant publications |
| --- | --- | --- | --- |
| JSTOR | Journal of New Music Research | European Journal of Sport Science | ScienceDirect |
| UCL Press | Arts and Health | Human Movement Science | Google Scholar |
|  | Music Perception | International Biomechanics | Royal College of Music Library |
|  | Biology of Sport | Journal of Biomechanical Science and Engineering | Imperial College London Library |
|  | Annals Kinesiologiae | Journal of Electromyography and Kinesiology | King’s College London Library |
|  | Journal of Functional Kinesiology | Journal of Biomechanics | The British Library |
|  | Journal of Science and Medicine in Sport | Journal of Human Kinetics | BioRxiv |
|  | Journal of Sport Biomechanics | Journal of Sports Sciences | CAB Abstracts |
|  | Science & Sports | Perceptual and Motor Skills | Cambridge Journals Online |
|  | Acta Biologica Bellica | Brain and Cognition | China Academic Journals |
|  | Activitas Nervosa Superior | Somatosensory and Motor Research | China/Asia On-Demand |
|  | Adaptive Human Behavior and Physiology | Journal of Neuroscience | Cochrane Library |
|  | Advances in Neuroscience | Journal of Neurophysiology | EBSCO |
|  | AIMS Neuroscience | Music & Science | Book Citation Index (Sciences) |
|  | AJOB Neuroscience | Neurophysiology | DART-Europe |
|  | Annals of Neurosciences | Applied Bionics and Biomechanics | ProQuest Dissertations & Theses |
|  | Annual Review of Neuroscience | Biology of Sport | Directory of Open Access Books |
|  | Behavioural Brain Research | European Journal of Applied Physiology | Directory of Open Access Journals |
|  | Biological Cybernetics | Clinical Biomechanics | Hathi Trust |
|  | BMC Neuroscience | Sports Biomechanics | Karger |
|  | Brain and Development | Frontiers in Human Neuroscience | Open Access Theses and Dissertations |
|  | Brain and Neuroscience Advances | Frontiers in Psychology | OpenDissertations |
|  | Brain, Behavior, and Evolution |  | Oxford Journals Online |
|  | Brain: Broad Research In |  | RIPM |
|  | Brain Multiphysics |  | RILM |
|  | Brain Plasticity |  | SAGE Journals |
|  | Brain Research |  | Science Citation Index Expanded |
|  | Brain Research Bulletin |  | SocArXiv |
|  | Brain Research Reviews |  | Taylor & Francis |
|  | Brain Sciences |  | Wiley Online Library |
|  | Clinical Neurophysiology |  | World Scientific Journals |
|  | Clinical Neurophysiology Practice |  | WorldCat |
|  | Cognitive Brain Research |  | WorldWideScience |
|  | Developmental Brain Research |  | Zetoc |
|  | Frontiers in Behavioural Neuroscience |  |  |
|  | Frontiers in Integrative Neuroscience |  |  |
|  | Reviews in the Neurosciences |  |  |
|  | Physiology and Behavior |  |  |
|  | Personality and Neuroscience |  |  |
|  | The Neuroscientist |  |  |
|  | Neuroscience Research |  |  |
|  | Neuroscience Letters |  |  |
|  | Neuroscience Bulletin |  |  |
|  | Neuroscience Insights |  |  |
|  | Neuroscience Journal |  |  |
|  | Neuroscience and Neurobehavioral Reviews |  |  |
|  | Neuroscience and Behavioural Physiology |  |  |
|  | Neuroscience |  |  |
|  | International Journal of Neuroscience |  |  |
|  | International Journal of Psychophysiology |  |  |
|  | Journal of Clinical Neurophysiology |  |  |
|  | Journal of Cognitive Neuroscience |  |  |
|  | Journal of Neuroscience |  |  |
|  | Journal of Neuroscience, Psychology, and Economics |  |  |
|  | Journal of Neuroscience Research |  |  |
|  | Journal of Psychophysiology |  |  |
|  | Journal of the Neurological Sciences |  |  |
|  | Nature Reviews Neuroscience |  |  |
|  | Nature Neuroscience |  |  |
|  | Frontiers in Bioengineering and Biotechnology |  |  |
|  | Acta Facultatis Educationis Fisicae Universitatis Comenianae |  |  |
|  | Acta Gymnica |  |  |
|  | Acta Universitatis Carolinae: Kinanthropologica |  |  |
|  | Annales Kinesiologiae |  |  |
|  | Biomedical Human Kinetics |  |  |
|  | BMJ Open Sport & Exercise Medicine |  |  |
|  | European Review of Aging and Physical Activity |  |  |
|  | European Journal of Sport Science |  |  |
|  | Adaptive Human Behavior and Physiology |  |  |
|  | Exercise and Sport Sciences Reviews |  |  |
|  | Human Movement |  |  |
|  | International Biomechanics |  |  |
|  | International Journal of Kinesiology in Higher Education |  |  |
|  | Journal of Sport Biomechanics (Arabic) |  |  |
|  | Frontiers in Neurology |  |  |
|  | Frontiers in Physiology |  |  |
|  | Frontiers in Neuroscience |  |  |
|  | International Journal of Performance Analysis in Sport |  |  |
